# Exosomes as Efficient Nanocarriers in Osteosarcoma: Biological Functions and Potential Clinical Applications

**DOI:** 10.3389/fcell.2021.737314

**Published:** 2021-10-12

**Authors:** Lingkai Yang, Xin Huang, Haoyu Guo, Lutong Wang, Wenbo Yang, Wei Wu, Doudou Jing, Zengwu Shao

**Affiliations:** Department of Orthopaedics, Union Hospital, Tongji Medical College, Huazhong University of Science and Technology, Wuhan, China

**Keywords:** exosomes, osteosarcoma, biomarker, targeted delivery, tumor microenvironment

## Abstract

Osteosarcoma is the most common bone tumor affecting both adolescents and children. Although localized osteosarcoma has an overall survival of >70% in the clinic, metastatic, refractory, and recurrent osteosarcoma have poorer survival rates. Exosomes are extracellular vesicles released by cells and originally thought to be a way for cells to discard unwanted products. Currently, exosomes have been reported to be involved in intercellular cross-talk and induce changes in cellular behavior by transferring cargoes (proteins, DNA, RNA, and lipids) between cells. Exosomes regulate osteosarcoma progression, and processes such as tumorigenesis, proliferation, metastasis, angiogenesis, immune evasion, and drug resistance. Increasing evidences shows that exosomes have significant potential in promoting osteosarcoma progression and development. In this review, we describe the current research status of exosomes in osteosarcoma, focusing on the biological functions of osteosarcoma exosomes as well as their application in osteosarcoma as diagnostic biomarkers and therapeutic targets.

## Introduction

Bone malignancies are the third leading cause of cancer-related deaths in children and adolescents (Jemal et al., 2007). Osteosarcoma is the primary bone malignancy that mainly affects this age group, accounting for about 60% of bone malignancies (Geller and Gorlick, 2010). Although localized osteosarcoma has an overall survival of >70% in the clinic, metastatic, refractory, and recurrent osteosarcoma have poorer survival rates. Thus, in this article, we mainly discuss the osteosarcoma with poor survival. The most common complication of osteosarcoma is the development of metastatic diseases (Marina et al., 2004). The lung is the most common site of metastasis accounting for >85% of metastatic disease, followed by the bone being the second most common site of distant metastasis (Isakoff et al., 2015). Lung metastases are the leading cause of death in over 30% of patients with osteosarcoma (Botter et al., 2014). The overall survival rate of metastatic osteosarcoma is 20% after 5 years and the prognosis is poor (Allison et al., 2012; Zhu et al., 2013; Friebele et al., 2015; Saraf et al., 2018). Therefore, early diagnosis is of great importance for increasing the long-term survival of patients, and it is urgent to find biomarkers with high sensitivity and specificity suitable for timely detection. For metastatic osteosarcoma, chemotherapy is the primary treatment, and drug resistance is common; thus, there is also a need for new approaches to improve the efficacy of chemotherapeutic agents and minimize their toxic side effects.

Nanodrug delivery systems have the potential to enhance and maintain the clinical efficacy of chemotherapeutic drugs with less side effects by improving the absorption, penetration, and distribution of chemotherapeutic drugs, and has been widely studied in tumor targeted drug therapy in recent years (Li et al., 2018; Rodríguez-Nogales et al., 2018; Tu et al., 2018). The secretion mode of exosomes, their good biocompatibility, and their nanoscale size makes them excellent potential nano-drug delivery carriers, which will be described in detail below.

Exosomes are widely accepted generic terms for naturally secreted lipid bilayer particles in cells, such as endogenous exosomes and plasma membrane derived exosomes (Théry et al., 2018). Exosomes contain transmembrane proteins and enveloped cytoplasmic nucleic acids, lipids, and proteins, which ultimately form a lipid bilayer that supports exosomes structure (Fu et al., 2019). Exosomes are very small, 30–150 nm vesicles, which have been proven to exert important communication between cells and are easily released by a variety of cells *in vivo* and *in vitro*, including cancer cells and normal cells (Keller et al., 2006). Exosomes can also be found in many body fluids, including blood, urine, semen, breast milk, saliva, amniotic fluid, and ascites (Xu et al., 2018). In addition, exosomes have been described as a source of biomarkers and are involved in various physiological processes including blood coagulation, angiogenesis, wound healing, tissue regeneration, pregnancy, autophagy, immune regulation, stem cell differentiation, and cancer progression (Wolf-Dennen and Kleinerman, 2020). Further, exosomes are important agents in metastasis, tumor growth, tumorigenesis, and angiogenesis (Peinado et al., 2012). Exosomes have been shown to contain a variety of cargoes, such as proteins, mRNAs, miRNAs, and single- and double-stranded DNA (Vlassov et al., 2012), which can reflect the identity of the cell of origin (Fu et al., 2019). Once released, exosomes can transmit multimolecular information to neighboring cells and distant targets through the extracellular fluid, thereby regulating their roles (Cappariello and Rucci, 2019).

The purpose of this review is to discuss the relationship between exosomes and osteosarcoma, focusing on the functions, mechanisms of action, and potential clinical applications of exosomes and their cargoes as biomarkers and therapeutic targets for osteosarcoma. But overall, the applicability of osteosarcoma derived exosomes can be used for all osteosarcoma subsets. Although there have been two recent reviews summarizing the relationship between exosomes and osteosarcoma, the first described only the role and application prospects of exosome derived non-coding RNAs for osteosarcoma, while the other focused more on exosome derived cargoes in osteosarcoma patients (Li and Wang, 2021; Zhang X. B. et al., 2021). This review provides a more comprehensive description of the function and application prospects of osteosarcoma exosomes, including not only as exosome cargoes in osteosarcoma, but also as carriers in osteosarcoma. In addition, all studies describing the relationship between exosomes and osteosarcoma were included.

## The Biogenesis and Uptake of Exosomes

**FIGURE 1:**
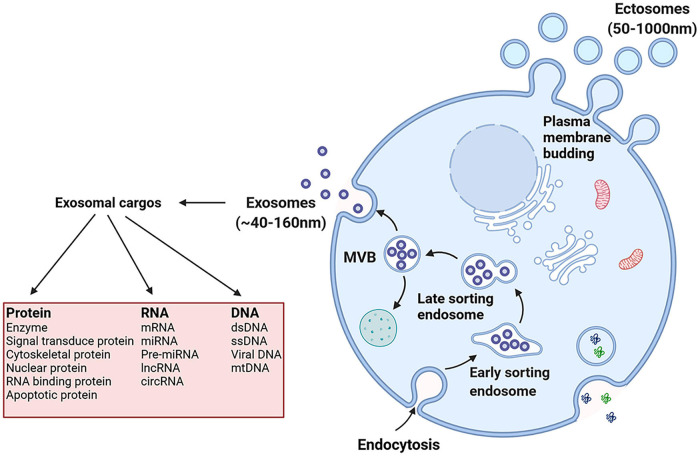
Exosomes originate from the endosomal pathway by the formation of the early sorting endosome (ESEs), late sorting endosome (LSEs), and ultimately multivesicular bodies (MVBs), which contain intraluminal vesicles (ILVs). Then MVBs fuse with the plasma membrane and exosomes are released. And exosomal cargoes include various types of biological molecules, such as protein, RNA and DNA.

The production of exosomes involves the double invagination of the plasma membrane and the formation of intracellular multivesicular bodies (MVBs) containing intraluminal vesicles (ILVs) (Kalluri and LeBleu, 2020). Early endosomes are formed when cell-surface proteins and soluble proteins are endocytosed via inward budding of the plasma membrane, which indicates exosome formation has initiated (Merchant et al., 2017; Kalluri and LeBleu, 2020). Early endosomes also fuse with the endoplasmic reticulum (ER), trans-Golgi network (TGN), and mitochondria (Kalluri, 2016; Hessvik and Llorente, 2018; van Niel et al., 2018; Willms et al., 2018; Mathieu et al., 2019; McAndrews and Kalluri, 2019). Early endosomes mature into late endosomes and eventually generate MVBs containing numerous ILVs formed by the emboly of the endosomal membrane (Figure 1; Merchant et al., 2017). During emboly, ILVs incorporate cytosolic and membrane proteins, lipids, and RNAs (Raiborg and Stenmark, 2009; van Niel et al., 2018). The MVBs can be degraded or fuse with the plasma membrane and by fusing with lysosomes or autophagosomes they release the contained ILVs as exosomes (Raiborg and Stenmark, 2009; Kahlert and Kalluri, 2013; Lo Cicero et al., 2015; van Niel et al., 2018). Furthermore, exosomes could also be formed through plasma membrane (PM) directly. The main difference between the MVB-dependent secretion of exosomes and PM derived exosomes is the different molecular mechanisms involved in exosomes formation. The former is associated with the endosomal sorting complex required for the transport complex (ESCRT) family, lipids, tetraspanins, and the Ras-related proteins in the brain (RAB) family (including RAB11, RAB35, RAB7, and RAB27), and in turn the RAB act on different MVBs. The MVB pathway is associated with proteins of ADP-ribosylation factor 6 (ARF6), and part of the ESCRT family [including ALG2-interacting protein X (ALIX) and tumor susceptibility gene 101 (TSG101)] (Colombo et al., 2014). Nonetheless, the different extracellular fate of these two types of exosomes is currently unknown and may be related to the cargoes they derive.

When exosomes arrive at the surface of receptor cells then may induce intracellular signals by binding to surface receptors (Denzer et al., 2000; Miyanishi et al., 2007). Exosome uptake has been linked to multiple mechanisms, including macropinocytosis, phagocytosis, clathrin-dependent endocytosis, and clathrin-independent endocytosis (Mulcahy et al., 2014; van Dongen et al., 2016). The different types of endocytosis are the primary methods of exosome uptake. However, which type of endocytic mechanism is involved in osteosarcoma and is the most important has little consensus in research and differences in exosome uptake reveal the heterogeneity of exosomes and the original cells (Mulcahy et al., 2014). Thus, it is important to explore this problem in future studies. Furthermore, exosomes, by fusing with the plasma membrane, directly release exosome cargoes into the cytoplasm of the recipient cell where they play subsequent biological roles (Parolini et al., 2009).

## The Functions of Exosomes in Osteosarcoma

Recent evidence has shown that exosomes play a vital role in the tumorigenesis, proliferation, metastasis, anti-apoptosis, immune evasion and chemoresistance of osteosarcoma. Garimella et al. first reported the function of exosomes in osteosarcoma. They detected factors such as MMPs, RANKL, TGFβ, and CD-9 in the exosomes of osteosarcoma cells, as well as changes in intracellular calcium concentration that can affect exosome biogenesis. They suggested a novel function of exosomes in driving bone resorption of osteoclastic by disrupting bone remodeling homeostasis in the osteosarcoma bone microenvironment (BME) (Garimella et al., 2014). This study opened a novel area in osteosarcoma research, linking emerging nanocarrier exosomes to the progression, treatment, prognosis, and even pathogenesis of osteosarcoma.

### Exosomes and Metastasis

Exosomes can mediate invasion and metastasis of osteosarcoma to local or distant organs and tissues. Groundbreaking research by Macklin et al. showed that exosomes from highly metastatic osteosarcoma cell subpopulations may increase invasion and chemotaxis of poorly metastatic osteosarcoma cell subpopulations. The authors concluded that highly metastatic clonal variants of osteosarcoma derived exosomes may drive metastatic behavior through cooperation between clones and preferential lung colonization (Macklin et al., 2016). More recently, Urciuoli et al. demonstrated that osteosarcoma cells can release transforming exosomes, which are responsible for transforming normal recipient cells into tumor-like phenotypes involving proliferation, metastasis, and antiapoptotic properties (Urciuoli et al., 2018). Mazumdar et al. demonstrated that exosomes derived from osteosarcoma cells can induce lung fibroblast reprogramming and thereby direct fibroblast activation and differentiation toward a myofibroblast/cancer-associated stromal fibroblasts (CAFs) phenotype through exosome-associated TGFβ1 and SMAD2 pathway activation. This study suggested that osteosarcoma derived exosomes could represent novel regulators of lung fibroblast activation, which may further explain how osteosarcoma cells regulate distant cells to potentially promote metastasis (Mazumdar et al., 2020). Cheng et al. identified that osteosarcoma derived exosomes induced M2 type polarization of macrophages through regulation of the expression of T cell immunoglobulin and mucin domain 3 (Tim-3) to promote the migration, invasion, epithelial mesenchymal transition (EMT), and distant metastasis of osteosarcoma cells (Cheng et al., 2021). In a subsequent study, Lagerweij et al. suggested that osteosarcoma-secreted exosomes strongly promote mesenchymal stem/stromal cells (MSCs) proliferation and metastasis by activating the IL-6/STAT3 signaling pathway (Lagerweij et al., 2018). Thus, altogether, these osteosarcoma derived exosomes may promote osteosarcoma metastasis to local or distant tissues.

### Exosomes and Tumor Microenvironments

MSCs are known as the host of the tumor microenvironments (TME) and take part in forming the TME as well as interacting with tumor cells (Karp and Leng Teo, 2009; Roorda et al., 2009). As novel carriers or intercellular communication tools, exosomes may play a potential role in the process depending on the cargoes they carry. Vallabhaneni et al. demonstrated that cell-to-cell communication via exosomes from stressed mesenchymal stem cells (SD-MSCs) can significantly affect the metastatic potential of osteosarcoma cells, which is strongly associated with the miRNA content of exosomes (Vallabhaneni et al., 2016). A series of studies have shown that human bone marrow MSCs (hBMSCs) and osteosarcoma cells can interact with each other using exosomes to deliver cargoes. A previous study by Qi et al. demonstrated that exosomes from hBMSCs act as paracrine factors for reactivation of the Hedgehog signaling pathway in osteosarcoma cells, suggesting that Hedgehog signaling plays an important role in the proliferation and metastasis of osteosarcoma (Qi et al., 2017). Subsequently, Lin et al. reported that through the PI3K/AKT pathway, exosomes from hBMSCs increase the expression of HIF-1α and its target genes and further promote osteosarcoma progression (Lin et al., 2019). Huang et al. demonstrated that exosomes from hBMSCs promote tumorigenesis and metastasis in osteosarcoma progression by promoting oncogenic autophagy both *in vitro* and *in vivo* (Huang et al., 2020). Thus, exosomes secreted by MSCs can influence the phenotype of osteosarcoma cells by acting as a regulatory factor in the TME.

### Exosomes and Immune Evasion

Another important feature of exosomes derived from osteosarcoma is their capacity to induce immune evasion. Troyer et al. demonstrated that exosomes secreted by malignant cells contain a unique cargo that can promote the differentiation of CD4 + cells to the T regulatory phenotype (CD4 +, CD25 +, FOXP3 +), and eventually leading to immune evasion of the malignant cells (Troyer et al., 2017). In a subsequent study, Brady et al. separated exosomes from the serum of healthy, fractured, and osteosarcoma-diagnosed dogs, and subjected these to proteomic analysis and found a mechanism of immune evasion in early and late stages of osteosarcoma. In exosomes of osteosarcoma-bearing dogs, expression of the plasma protease C1 inhibitor increased and the expression of C1qa decreased, which could prevent the activation of the classical pathway as a potential evasion mechanism of osteosarcomas in ascitic fluids and in the serum of osteosarcoma patients (Brady et al., 2018). More recently, Wolf-Dennen et al. demonstrated that metastatic osteosarcoma cell derived exosomes can regulate cellular signaling of TAMs to promote the M2 phenotype and create an immunosuppressive, tumor-promoting microenvironment through the production of TGFβ2 (Wolf-Dennen et al., 2020). To some extent, these studies explain why osteosarcoma has such a poor prognosis as a malignant tumor.

### Exosomes and Chemotherapy Resistance

Exosomes can also induce osteosarcoma chemotherapy resistance. Torreggiani et al. reported that multidrug-resistant osteosarcoma cells are able to transfer osteosarcoma derived exosomes carrying MDR-1 mRNA and its product P-glycoprotein so as to spread their resistance to doxorubicin treatment on sensitive osteosarcoma cells (Torreggiani et al., 2016). Pan et al. demonstrated that the resistance of chemotherapy-sensitive osteosarcoma cells to cisplatin (cis-diamminedichloroplatinum, CDDP) could be enhanced through CDDP drug-resistant cell derived exosomal hsa_circ_103801 (Pan et al., 2021). The above studies may partially explain the mechanism of drug resistance in chemotherapy-sensitive osteosarcoma cells, and may also provide a new mechanism for the selection of chemotherapy drugs for osteosarcoma patients.

## The Functions of Cargoes Derived From Exosomes in Osteosarcoma

In addition to exosomes, exosomal cargoes can also have direct or indirect effects on the biological function of osteosarcoma cells or other cells. Exosomes-delivered bioactive molecules result in the intercellular exchange of genetic information and recipient cell reprogramming (Fu et al., 2019). Exosome derived cargoes can be used to clarify biogenesis processes, recognize parent cells, and regulate biological functions (Tang and Wong, 2015). Below, we will discuss exosomal cargoes carried by exosomes derived from osteosarcoma cells and other cells in the TME, including exosomal RNAs [such as Micro RNAs (miRNAs), Long non-coding RNAs (lncRNAs), and Circular RNAs (circRNAs)] and proteins (Table 1).

**TABLE 1:** Overview of exosomal cargoes and functions in osteosarcoma.

**Cargo** **type**	**Exosomal cargo**	**Cell derived exosome source**	**Recipient cells**	**Function**	**References**
miRNA	miR-675	143B and Well5	hFOB 1.19	Promote cell migration and invasion	Gong et al., 2018
	miR-25-3p	143B and U2OS	Human umbilical vein endothelial cell	Induce cell formation and migration	Yoshida et al., 2018
	miR-143	MSC	143B	Reduce cell migration	Shimbo et al., 2014
	miR-1228	CAF	MG63 and HOS	Promote cell migration and invasion	Wang Y. et al., 2019a
	miR-208a	BMSC	MG62 and Saos-2	Promote cell migration and invasion	Qin et al., 2020
	miR-101	ADSC	143B and Saos-2	Suppress cell migration and invasion	Zhang et al., 2020c
	miR-206	BMSC	143B	Inhibit cell proliferation, migration, invasion and induce apoptosis	Zhang et al., 2020a
lncRNA	PVT1	BMSC	Saos-2, MG63 and MNNG/HOS	Promote tumor growth and metastasis	Zhao et al., 2019
	linc00852	143B and HOS	143B and HOS	Promote tumor growth, invasion and metastasis	Li Q. et al., 2020
	LIFR-AS1	Macrophage	143B and U2OS	Promote tumor proliferation, invasion and restrain cell apoptosis	Zhang H. et al., 2021
	CASC15	143B and U2OS	143B and U2OS	Promote tumor growth and metastasis	Zhang et al., 2020b
	EWSAT1	HOS	143B, U2OS and MG63	Promote tumor proliferation, migration and survival	Tao et al., 2020
circRNA	circ-0000190	hFOB1.19	MG63 and HOS	Reduce cell proliferation, invasion and migration	Li S. et al., 2020
	hsa-circ-103801	MG63/CDDP	MG63 and U2OS	Reduce sensitivity to CDDP and inhibit apoptosis	Pan et al., 2021
Protein	uPA	BMSC and KHOS	KHOS	Promote cell migration, invasion and metastasis	Endo-Munoz et al., 2015
	TGFβ	143B	MSC	Promote tumor growth and metastasis	Baglio et al., 2017
	COLGALT2	ADSC	U2OS and MG63	Promote tumor growth and metastasis	Wang Y. et al., 2020
	COL6A1	U2OS and MG63	Normal fibroblasts	Promote tumor invasion and migration	Zhang Y. et al., 2021
	Tim-3	MG63	Macrophages	Promote tumor invasion and metastasis	Cheng et al., 2021
	PD-L1 and N-cadherin	143B	143B and U2OS	Promote tumor pulmonary metastasis	Wang J. et al., 2020
	Hic-5	MG63	MG63 and HOS	Regulate tumor apoptosis and proliferation	Sha et al., 2020
	LCP1	BMSC	143B and HOS	Promote tumor metastasis and proliferation	Ge et al., 2020

*MSC, mesenchymal stem/stromal cell; CAF, cancer-associated stromal fibroblast; BMSC, bone mesenchymal stromal cell; ADSC, adipose tissue-derived mesenchymal stromal cell; CASC15, cancer susceptibility 15; MG63/CDDP, cisplatin-resistant MG63; uPA, urokinase plasminogen activator; COLGALT2, collagen beta galactosyl transferase 2; COL6A1, Collagen type VI alpha 1; Tim-3, T cell immunoglobulin and mucin domain 3; PD-L1, programmed death-ligand 1;Hic-5, hydrogen peroxide inducible clone 5; LCP1, lymphocyte cytosolic protein 1.*

### Exosomes Derived RNAs in Osteosarcoma

#### Exosomes Derived miRNAs in Osteosarcoma

Many studies have investigated the existence of osteosarcoma exosome derived genetic material (including DNA and RNA) and how these specifically affect downstream biological processes. Currently, the best described among these molecules are miRNAs. The existence of exosome derived miRNAs in a series of human osteosarcoma or osteoblastic cell lines with different levels of metastatic potential was identified by next generation miRNA sequencing as reported by Jerez et al. The most notable miRNAs are miR-21-5p, miR-143-3p, miR-148a-3p, and miR-181a-5p. Gene ontology analysis of predicted target genes for these miRNAs suggested that they control proteins that regulate cell apoptosis, angiogenesis, adhesion, and migration (Jerez et al., 2019). Ye et al. purified exosome-like vesicles from the plasma of patients with osteosarcoma and healthy controls, and following high-throughput sequencing verified that 57 miRNAs were differentially expressed, 20 of which were upregulated and 37 downregulated. Studies *in vitro* and *in vivo* have shown that exosomes secreted from miR-195-3p upregulated osteosarcoma cells promote cell proliferation and invasion via exosomal miR-195-3p (Ye et al., 2020). Han et al. reported that osteosarcoma cell derived exosomal miR-1307 targets AGAP1, which is an Arf GTPase-activating proteins (Arf GAPs) that depends on phosphoinositide to promote the proliferation, invasion and migration of osteosarcoma cells, while upregulation of AGAP1 could significantly inhibit the function of miR-1307 in osteosarcoma (Han et al., 2021).

Osteosarcoma cells-secreted exosomes can also transfer miRNAs to other cells in the TME. Gong et al. observed that invasion and migration of non-malignant osteoblast cells (hFOB 1.19) were increased by exposure to exosomes derived from metastatic osteosarcoma by delivering exogenous miR-675 and downregulating target genes within recipient cells such as CALN1 (Gong et al., 2018). Yoshida et al. also reported that extracellular miR-25-3p enriched in osteosarcoma derived exosomes are transported to endothelial cells and stimulated angiogenesis (Yoshida et al., 2018).

Emerging studies have suggested that during osteosarcoma progression, miRNAs could be delivered by non-tumor cells derived exosomes, such as MSCs, CAFs and adipose tissue derived mesenchymal stromal cells (ADSCs), to osteosarcoma cells. Shimbo et al. demonstrated that introducing synthetic miR-143 into MSCs could increase MSCs secreted exosomes and the amount of exosome-formed miR-143 in the conditioned medium, which could easily be transferred into recipient cells and inhibit the migration of the 143B osteosarcoma cell line (Shimbo et al., 2014). Qin et al. demonstrated that bone mesenchymal stromal cell (BMSC) derived exosomal miR-208a negatively targeted PDCD4 to activate the ERK1/2 signaling pathway, thereby promoting proliferation, migration, and invasion of osteosarcoma cells (Qin et al., 2020). More recently, Zhang et al. demonstrated that by targeting TRA2B, exosome derived miR-206 derived from BMSC could be transferred into osteosarcoma cells and suppress osteosarcoma progression (Zhang et al., 2020a). Wang et al. reported that CAFs can transfer miR-1228 into human osteosarcoma cells via CAFs-secreted exosomes, ultimately promoting migration and invasion of the osteosarcoma cells *in vitro* (Wang J. W. et al., 2019). The study by Zhang et al. revealed that ADSCs derived miR-101-enriched exosomes showed suppressive effects on osteosarcoma cell invasion and migration via downregulation of B cell lymphoma 6 (BCL6) (Zhang et al., 2020c). These could provide a new approach for the treatment of osteosarcoma, which involves other cells secreting exosomes carrying specific cargoes to inhibit the progression of osteosarcoma and thus take certain therapeutic effects on osteosarcoma cells.

#### Exosome Derived lncRNAs and circRNAs in Osteosarcoma

**FIGURE 2:**
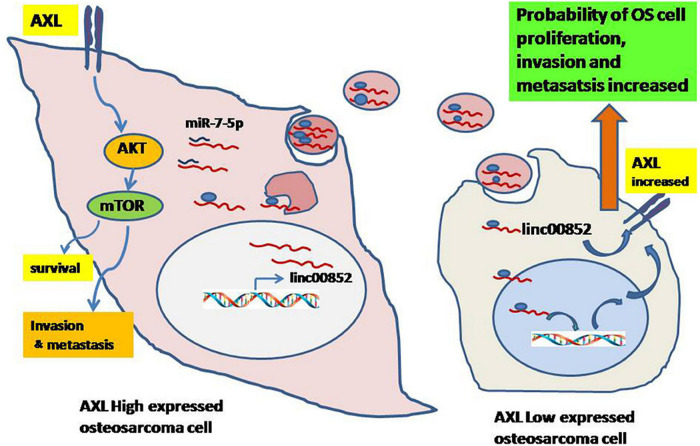
Schematic diagram of exosomal linc00852 function in the growth and progression of osteosarcoma. Osteosarcoma cells with high AXL expression secrete exosomal linc00852 and then are transmitted to cells with low AXL expression. Most linc00852 transfer to receptor cells’ nucleus, increase AXL expression to activate AKT pathway and promote osteosarcoma progression with unknown mechanisms. Meanwhile, a few linc00852 play a role as ceRNA for miR-7-5p to increase expression of AXL in cytoplasm. Reprinted from Li Q. et al. (2020).

In addition to miRNAs, exosomes also contain several other series of non-coding RNAs, such as circRNAs and lncRNAs, which also play important roles in osteosarcoma. Groundbreaking research by Zhao et al. revealed that by transporting lncRNA PVT1 to osteosarcoma cells, exosomes derived from BMSC could promote osteosarcoma metastasis and growth (Zhao et al., 2019). In addition, upregulation of plasmacytoma variant translocation 1 (PVT1) stabilizes ERG protein by inhibiting ERG ubiquitination, and upregulation of ERG expression by competitive binding with miR-183-5p (Zhao et al., 2019). Li et al. demonstrated that osteosarcoma could releasing exosomes that contain linc00852 and there is a positive feedback regulation loop between AXL and exosome derived linc00852. As a result, the growth, invasion and metastasis of osteosarcoma cells with low AXL expression were able to be promoted by osteosarcoma cells with high AXL expression (Figure 2; Li Q. et al., 2020). Zhang et al. reported that macrophages derived exosomal lncRNA LIFR-AS1 via the miR-29a/NFIA axis promoted osteosarcoma cell proliferation and invasion (Zhang H. et al., 2021). Pan et al. demonstrated that CDDP drug-resistant osteosarcoma cell derived exosomes could transfer hsa_circ_103801 to chemotherapy-sensitive osteosarcoma cells to enhance the resistance of osteosarcoma cells to CDDP (Pan et al., 2021). Zhang et al. demonstrated that lncRNA cancer susceptibility 15 (CASC15) is upregulated in osteosarcoma plasma exosomes, and by targeting the miR-338-3p/RAB14 axis, downregulation of CASC15 inhibited the progression of osteosarcoma including proliferation, migration, and invasion (Zhang et al., 2020b). Tao et al. reported that Ewing sarcoma associated transcript 1 (EWSAT1; also known as linc-00277) regulated osteosarcoma-induced angiogenesis via a “double stacking effect,” through which exosomes-carrying EWSAT1-induced increased sensitivity of vascular endothelial cells and EWSAT1-induced increased secretion of angiogenic factors in osteosarcoma cells, and by transporting EWSAT1 from osteosarcoma cells to vascular endothelial cells, these exosomes play a crucial role in this process (Tao et al., 2020). Li et al. recently identified circ-0000190 in osteosarcoma microarray, which was found to be down-regulated in osteosarcoma cell lines. The study reported that normal cells could transport circ-0000190 to osteosarcoma cells via secreted exosomes, and regulated TET1 expression by competitive binding with miR-767-5p, thus significantly reducing the proliferation, invasion, and migration of cancer cells (Li S. et al., 2020).

### Exosome Derived Proteins in Osteosarcoma

Proteins are also one of the main components of exosomes. Exosomes exhibit specific proteins that depend on the type of secretory cell, and also carry specific subsets of cell proteins that are independent of cell type (He et al., 2018). These proteins are implicated in some basic cellular processes, such as structural dynamics, cell adhesion, metabolism, membrane fusion, and signal transduction (Simpson et al., 2009). However, some proteins are involved in osteosarcoma progression and play important roles in osteosarcoma growth, migration, invasion, and metastasis. To date, several exosomes derived proteins have been shown to be involved in osteosarcoma progression.

**FIGURE 3:**
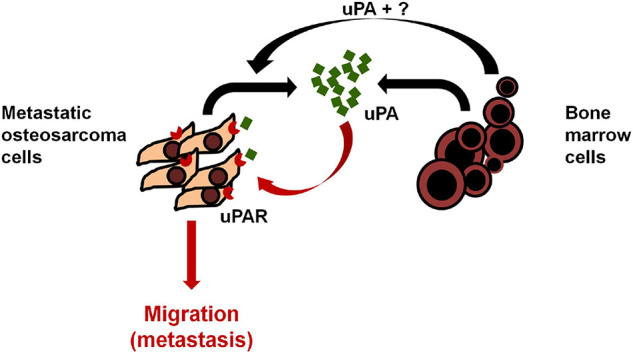
Model of uPA/uPAR signaling in osteosarcoma and the bone microenvironment. Osteosarcoma cells secrete uPA into the surrounding microenvironment which acts in an autocrine fashion by binding to osteosarcoma uPAR and promoting osteosarcoma cell migration, invasion and metastasis. Bone marrow cells in the surrounding microenvironment promote the expression of uPA/uPAR in osteosarcoma cells in a paracrine manner to increase metastasis. Reprinted from Endo-Munoz et al. (2015).

**FIGURE 4:**
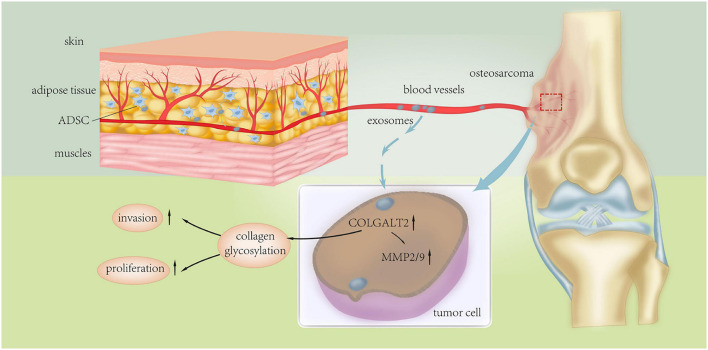
Schematic diagram of the underlying mechanism of ADSC exosomes in osteosarcoma proliferation and metastasis. Adipose tissue-derived mesenchymal stromal cell (ADSC) exosomes induce upregulation of COLGALT2 in osteosarcoma cells, accompanied by increased expression of vimentin and MMP2/9. Reprinted from Wang Y. et al. (2020).

Endo-Munoz et al. demonstrated that metastatic osteosarcoma cells can promote metastasis via locally secreted urokinase plasminogen activator (uPA) and via uPA-containing osteosarcoma derived exosomes at distant sites (Figure 3; Endo-Munoz et al., 2015). Baglio et al. reported that osteosarcoma-secreted exosomes carry membrane-associated TGFβ to the surface of MSCs where TGFβ interacts with the ALKV receptor and modifies their behavior to promote osteosarcoma growth and metastasis formation (Baglio et al., 2017). More recently, Wang et al. demonstrated that ADSC-secreted exosomes can activate the expression of Homosapien collagen beta galactosyl transferase 2 (COLGALT2) in osteosarcoma cells, thereby increasing vimentin and MMP expression to promote osteosarcoma growth and metastasis (Figure 4; Wang Y. et al., 2020). Zong et al. demonstrated for the first time that osteosarcoma cells carrying the RAB22A-NeoF1 fusion gene acts as a metastasizing driver that may secrete exosomes containing RAB22A-NeoF1 into the osteosarcoma microenvironment, thereby affecting the characteristics of RAB22A-NeoF1-negative osteosarcoma cells. Further studies have shown that PYK2 was enriched in osteosarcoma derived exosomes by binding to the RAB22A-NeoF1 fusion protein, thereby inducing RhoA activation in RAB22A-NeoF1-negative receptor osteosarcoma cells to promote their migration, invasion, and lung metastasis (Zhong et al., 2021). Wang et al. also revealed that by releasing osteosarcoma derived exosomes carrying programmed death-ligand 1 (PD-L1) and N-cadherin, osteosarcoma could stimulate lung metastasis (Wang J. et al., 2020).

In addition to the direct roles of exosome derived proteins, there are also series of proteins that influence the characteristics of osteosarcoma through the regulation of downstream signaling pathways. Zhang et al. revealed that exosomal Collagen type VI alpha 1(COL6A1) derived from osteosarcoma cells promotes invasion and migration of osteosarcoma cells by secreting pro-inflammatory cytokines including IL-6 and IL-8, transforming normal fibroblasts into CAFs and mediating the TGF-β/COL6A1 signaling pathway (Zhang Y. et al., 2021). Ge et al. also demonstrated that through the JAK2/STAT3 pathway, BMSC derived exosomal lymphocyte cytosolic protein 1 (LCP1) could promote osteosarcoma proliferation and metastasis (Ge et al., 2020). Sha et al. proved that through inhibiting Wnt/β-catenin signals by the osteosarcoma derived exosome pathway, silencing hydrogen peroxide inducible clone 5 (Hic-5) prevented proliferation and promoted apoptosis of osteosarcoma cells (Sha et al., 2020).

To date, there have been many studies on osteosarcoma exosomes and their exosome derived cargoes, which have specifically described the progression and mechanism of osteosarcoma exosomes and exosomes derived cargoes. On this basis, some exosomes and their cargoes have been synthesized for clinical application in osteosarcoma and are expected to be used formally in clinical trials in the future.

### Exosome Derived DNAs in Osteosarcoma

DNAs are also one type of exosome derived cargoes and include dsDNA, ssDNA, viral DNA and mtDNA. To date, it is unknown whether specific DNAs will be sorted selectively into exosomes to play physiological roles in cells. Some studies have sequenced exosome-related DNAs and will provide the complete genome sequence of cells that secrete exosomes (Kahlert et al., 2014; Thakur et al., 2014). The distribution of exosome derived DNAs by these functional cells is not clear. Exosomes may play a role in inflammation regulation, or act as biomarkers of specific tumors, viral infection, or chemoresistance (Pegtel and Gould, 2019). Thus, the specific role of osteosarcoma derived exosomal DNAs in the progression of osteosarcoma and their clinical applications require further research.

## Potential Application of Exosomes in Osteosarcoma

### Exosomes as Biomarkers in Osteosarcoma

The accessibility and stability of exosomes in most body fluids make them a novel candidate for osteosarcoma liquid biopsy. A growing number of studies have shown that exosomes may have potential roles in early diagnosis, prognosis prediction, and efficacy evaluation of osteosarcoma (Table 2).

**TABLE 2:** Exosomes extracted from biofluids as diagnostic and prognostic biomarkers for osteosarcoma.

**Cargo** **type**	**Exosomal cargo**	**Existence**	**Extraction method**	**Identification method**	**Method**	**Clinical value in osteosarcoma**	**References**
miRNA	miR-675	Serum	Ultracentrifugation	TEM and Western blot	qRT-PCR	Biomarker for predicting the metastasis of OS	Gong et al., 2018
	miR-148a, miR-27a, miR-9 and miR-199a-3p	Serum	Differential centrifugation	Not shown	qRT-PCR	Diagnostic biomarkers for differential chemotherapeutic response to OS	Xu et al., 2017
	miR-25-3p	Extracellular fluid	Ultracentrifugation	SEM and Western blot	RT-PCR	Diagnostic biomarkers and indicate poor prognosis of OS patients	Yoshida et al., 2018
	miR-21-5p and miR-143-3p	Extracellular fluid	Ultracentrifugation	NTA	qRT-PCR	Elevated expression in OS and could be used as diagnostic biomarkers	Jerez et al., 2019
	miR-130a-3p and miR-195-3p	Plasma	Ultracentrifugation	TEM and Western blot	qRT-PCR	Elevated expression in OS and could be used as diagnostic biomarkers	Ye et al., 2020
	miR-101	Plasma	Differential centrifugation	Scanning confocal microscope and Western blot	qRT-PCR	Circulating biomarker for OS detection	Zhang et al., 2020c
lncRNA	linc00852	Extracellular fluid	Differential centrifugation	TEM and Western blot	qRT-PCR	Biomarker for OS	Li Q. et al., 2020
circRNA	circ-0000190	Plasma	Ultracentrifugation	TEM, Western blot and fluorescence microscope	qRT-PCR	Potential biomarker for OS detection	Li S. et al., 2020
	Has-circ-103801	Serum	Ultracentrifugation	TEM and Western blot	qRT-PCR	Effective prognostic biomarker for OS	Pan et al., 2021

*TEM, transmission electron microscope; qRT-PCR, quantitative real time polymerase chain reaction; OS, osteosarcoma; SEM, scanning electron microscope; NTA, nanoparticle tracking analysis.*

#### Exosomes Derived miRNAs as Biomarkers

Recent studies have demonstrated that exosome derived miRNAs may serve as effective biomarkers for osteosarcoma. Xu et al. reported that exosomal miRNAs in serum could be used as a reliable diagnostic biomarker for distinguishing differences in chemotherapy responses in osteosarcoma (Xu et al., 2017). A preliminary study by Bao et al. revealed the potential for metastatic biopsies using circulating exosomal RNAs as the sample (Bao et al., 2018). Gong et al. reported that lung metastasis leads to remarkably higher levels of circulating exosome derived miR-675 in patients with poorly progressing osteosarcoma and circulating exosome derived miR-675 was also related to CALN1 (a target gene of miR-675) expression in osteosarcoma tissues. The study demonstrated that serum exosome derived miR-675 expression may serve as a new biomarker for osteosarcoma metastasis (Gong et al., 2018). Cuscino et al. identified eight novel miRNAs in osteosarcoma cell lines and their release in exosomes, which demonstrated that these candidate miRNAs could potentially represent biomarkers for osteosarcoma (Cuscino et al., 2019). Yoshida et al. reported that the levels of exosomal miR-25-3p were significantly correlated with a poor prognosis of osteosarcoma (Yoshida et al., 2018). Jerez et al. showed that miR-21-5p and miR143-3p had higher expression levels in the exosomes of metastatic osteosarcoma cell lines, and thus could act as prognostic biomarkers for osteosarcoma (Jerez et al., 2019). In addition, Ye et al. also purified exosomal vesicles from plasma of patients with osteosarcoma and healthy controls and identified exosomal miRNAs with differential expression, demonstrating that these could be used as novel diagnostic biomarkers (Ye et al., 2020). More recently, Zhang et al. demonstrated that exosome derived miR-101 could become a hopeful circulating biomarker of osteosarcoma metastasis (Zhang et al., 2020c).

#### Exosomes Derived lncRNAs and circRNAs as Biomarkers

Exosome derived lncRNAs and circRNAs are also considered important diagnostic markers for osteosarcoma. Recently, Li et al. showed that the release of exosomes containing linc00852, contributed to the metastasis, invasion, and proliferation of osteosarcoma cells with low AXL expression and was promoted by osteosarcoma cells with high AXL expression. A positive feedback loop between exosome derived linc00852 and AXL demonstrated that exosome derived linc00852 may act a novel osteosarcoma biomarker (Li Q. et al., 2020). Growing evidence indicates that the expression of circRNAs in serum is correlated with prognosis, clinical severity, and metastasis of osteosarcoma and may also distinguish osteosarcoma from controls, which provides support for the utility of circRNAs as a biomarker for osteosarcoma (Li and Song, 2017; Kun-Peng et al., 2018a,b; Nie et al., 2018; Yin et al., 2018; Jin et al., 2019; Li S. et al., 2020). Additional evidence indicates that circRNAs are enriched and stabilized in exosomes (Wang Y. et al., 2019). A preliminary study by Pan et al. reported that the serum levels of exosomal hsa-circ-103801 were upregulated in osteosarcoma patients with a shorter survival time, indicating that high hsa-circ-103801 expression may serve as an effective prognostic biomarker for osteosarcoma (Pan et al., 2021).

#### Exosomes Derived Proteins as Biomarkers

In addition to exosome derived RNAs, exosome derived proteins can also serve as potential biomarkers for predicting prognosis in osteosarcoma patients. Wang et al. demonstrated that in exosomes from the serum of osteosarcoma patients PD-L1 and N-cadherin coexisted. The authors developed biomarkers of exosomal PD-L1 and N-cadherin from serum of osteosarcoma patients to predict pulmonary metastasis (Wang J. et al., 2020).

Therefore, exosome derived cargoes have the great potential to become novel diagnostic and prognostic biomarkers for osteosarcoma. We believe that exosomes and exosomes derived cargoes as biological tools will play an important role in the prediction and diagnosis of osteosarcoma soon. Then this application of exosomes could improve the survival of osteosarcoma patients which is important clinically for clinicians.

### Exosomes as Therapeutic Targets and Novel Modes of Drug Delivery in Osteosarcoma

#### Exosomes as Therapeutic Targets

The important functions of exosomes in osteosarcoma prove that it could also be developed as therapeutic targets. The osteosarcoma derived exosomal biomarkers mentioned above could also be utilized as therapeutic targets of osteosarcoma for the same origin of osteosarcoma cells. Baglio et al. reported that the osteosarcoma-secreted exosomes carry functional TGFβ molecules that increase IL-6 expression to promote osteosarcoma growth and metastasis formation, and the combination of IL-6 blocking agents with TGFβ inhibitors could stop osteosarcoma progression while reducing drug resistance (Baglio et al., 2017). More recently, Notaro et al. demonstrated that WIN (synthetic agonist of cannabinoid receptors) induced a significant increase in the number of secreted exosomes. Moreover, WIN-treated cells-isolated exosomes showed prominent anti-migratory effects in untreated osteosarcoma cells, which could be considered as a potential innovative therapeutic agent in osteosarcoma therapy (Notaro et al., 2019).

#### Exosomes Derived RNAs as Therapeutic Targets

Recent studies have also suggested that exosome derived RNAs may serve as therapeutic targets for osteosarcoma. Zhang et al. demonstrated that exosome derived miR-206 derived from BMSC can be transported into osteosarcoma cells and inhibit osteosarcoma progression via targeting TRA2B (Zhang et al., 2020a). A study by Zhang et al. showed that exosome derived miR-101 has metastatic inhibitory properties in osteosarcoma (Zhang et al., 2020c). Ye et al. identified several dysregulated exosome derived miRNAs in patients with osteosarcoma (Ye et al., 2020). Wang et al. showed that via downregulating mRNA expression of SCAI in osteosarcoma, the exosome derived miR-1228 could facilitate migration and invasion of osteosarcoma (Wang J. W. et al., 2019). Yoshida et al. reported that the expression of the WNT signaling inhibitor DKK3, an intracellular and extracellular miR-25-3p target, was correlated with a good prognosis in osteosarcoma patients (Yoshida et al., 2018). Shimbo et al. demonstrated that miR-143 derived from exosomes was easily transported into recipient cells and inhibited the migration of the 143B osteosarcoma cell line (Shimbo et al., 2014). Han et al. demonstrated that overexpression of AGAP1 could inhibit the function of exosomal miR-1307 in osteosarcoma (Han et al., 2021). Zhang et al. reported that exosomal lncRNA LIFR-AS1 promote osteosarcoma progression via the miR-29a/NFIA axis (Zhang H. et al., 2021). The above studies highlight novel therapeutic targets for osteosarcoma.

#### Exosomes Derived Proteins as Therapeutic Targets

In addition to exosomes derived RNAs, exosomes derived proteins can also act as biological therapeutic targets for the treatment of osteosarcoma patients. Cheng et al. identified osteosarcoma derived exosomes could induce M2 type polarization of macrophages by regulating Tim-3 expression (Cheng et al., 2021). Zhang et al. demonstrated that in plasma exosomes of osteosarcoma CASC15 is upregulated, and knockdown of CASC15 can inhibit osteosarcoma progression by targeting the miR-338-3p/RAB14 axis (Zhang et al., 2020b). Tao et al. designed precision drugs (EWSAT1-KD) targeting EWSAT1 to effectively target osteosarcoma cells (Tao et al., 2020). Ge et al. reported that BMSC derived exosomal LCP1 promotes osteosarcoma progression through the JAK2/STAT3 pathway, and miR-135a-5p could interact upstream of LCP1 to cancel tumorigenesis of osteosarcoma influenced by LCP1 (Ge et al., 2020). All this studies have provided evidence supporting these targets as a potential treatment of osteosarcoma patients.

#### Exosomes as Vehicles for Drugs

Exosomes can also be used to deliver chemotherapy drugs for osteosarcoma therapy, which is a better way to reduce the serious side effects of chemotherapy as well as prolong the half-life of chemotherapy drugs. Recently, Wei et al. reported that the exosome isolated from MSCs could be used as a drug nanocarrier to load chemotherapeutic drug doxorubicin, and Exo-doxorubicin could kill osteosarcoma cells more effectively when compared with free doxorubicin (Wei et al., 2019).

The application of exosomes as therapeutic targets and drug delivery vectors may provide a novel approach for the treatment of osteosarcoma patients. These targets are relevant and important for clinicians and patients in the clinic as they may contribute to improve survival of osteosarcoma patients.

## Discussion and Future Implications

**FIGURE 5:**
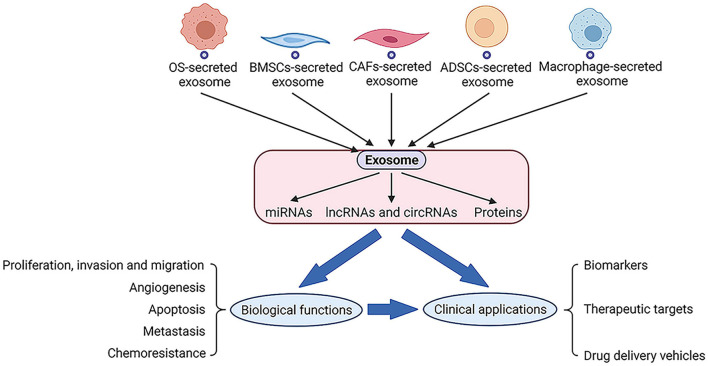
Exosomes could be secreted by series of cells and exosomal cargoes such as RNAs and proteins will perform biological functions along with exosomes, which may also lead to related clinical applications.

Exosomes could be secreted by different types of cells including osteosarcoma, BMSCs, CAFs, ADSCs, macrophages, and the secreted exosomes may contain a series of cargoes such as miRNAs, lncRNAs, circRNAs, and proteins. The exosomes and exosomal cargoes derived from these cells could exert biological activity and modify the functional activity of recipient cells, including proliferation, invasion, migration, angiogenesis, apoptosis, metastasis and chemoresistance. Moreover, several important branches are being explored to better define the clinical applications for exosomes and exosomal cargoes, including biomarkers, therapeutic targets and drug delivery vehicles (Figure 5).

Recent studies on exosomes have changed our understanding of the role of exosomes in osteosarcoma and provided new targets for the diagnosis and treatment of osteosarcoma. The role of exosomes in changing osteosarcoma behavior, remodeling TME and producing osteosarcoma drug resistance has attracted extensive attention. The existing studies of exosomes and their cargoes have partially unraveled the mechanism of action of osteosarcoma-associated exosomes. For example, there is molecular communication between osteosarcoma cells and MSCs that help establish a premetastatic niche in the TME. In addition, exosomes carrying specific cargoes in the circulation have potential diagnostic value, which reflects the progression and metastasis stages of osteosarcoma and predict the prognosis of osteosarcoma. Exosomes-based therapy has shown great promise. Blocking exosome-based cargo and its target or developing exosome-based drug delivery provides a novel strategy for the treatment of osteosarcoma.

Circulating exosomes derived cargoes have a great potential for early cancer detection, therapy, and prognosis. Nonetheless, using exosomes as biomarkers in diagnosis and prognosis, may result in false positive and negative findings due to the quality and heterogeneity of exosomes. Although the quality of exosomes has improved recently, and cancer therapy based on exosomes is supported by several early clinical trials. In pancreatic ductal adenocarcinoma (PDAC), engineered MSC derived exosomes carrying a commonly mutated gene KRAS siRNA is under research in early-phase clinical trial to treat metastatic pancreas cancer patients with the KRAS mutation (NCT03608631) (Kamerkar et al., 2017). The safety of dendritic cell (DC) derived exosomes for patients and the feasibility of producing DC derived exosomes are supported by early-phase clinical trials in cancer patients (Escudier et al., 2005; Morse et al., 2005). In colorectal cancer (CRC), tumor derived exosomes containing melanoma-related antigen recognized by T cells (Mart1) in ascites (Aex) was used to deliver Mart1 to DCs and Aex-containing exosomes plus granulocyte-macrophage colony-stimulating factor (GM-CSF) induced tumor-specific antitumor CTL response in early-phase clinical trials (Dai et al., 2008). In these three clinical trials, it is not difficult to appreciate that in addition to natural exosomes, engineered exosomes have been developed to meet the needs of clinical applications. There are two strategies used to produce engineered exosomes: direct extracellular modification and indirect intracellular modification. Extracellular modification often refers to the direct load of cargoes after exosomes isolation and purification, and intracellular modification means the parental cells are manipulated before exosomes release by physical or genetic approaches (Ma et al., 2020). However, the therapeutic potential of exosomes, the method of drug loading, the safety of exosomes as carrier, and the choice of exosomes doner cells are variables awaiting further investigation. A few exosome isolation techniques have been developed, including filtration, size-exclusion chromatography, ultracentrifugation, immunoaffinity capture, and microchip-based techniques (Zhang X. B. et al., 2021). However, the effects of different exosome isolation methods on the size, integrity, RNA, and protein cargoes of exosomes are unknown, which could lead to different sizes and concentrations of exosomes. Therefore, a standardized and stable isolation method is needed to produce exosomes for clinical application. Furthermore, the different storage temperatures of exosomes suitable for maintaining the vitality of exosomes in the clinical setting and in laboratory is also a significant challenge for clinicians (Park et al., 2018).

Although increasing attention has been paid to the application of exosomes in osteosarcoma, there are still several problems to be addressed before they can be used in the clinical treatment of osteosarcoma, including the specific mechanism of action of exosomes in osteosarcoma, isolation techniques, quantification, and the analysis of exosomes in body fluids of patients with osteosarcoma, as well as methods for detecting circulating osteosarcoma-specific exosomal cargoes (DNA, RNA, protein), and the preparation, storage and drug loading methods of engineered exosomes for treating osteosarcoma. In addition, there are many exosomes and exosomal cargoes awaiting to be discovered, providing more biological support for their role in the progression, treatment, and prognosis of osteosarcoma. Therefore, further efforts are needed to understand the role and mechanism of exosomes in osteosarcoma and to develop exosomes-based diagnosis, prognosis prediction and treatment options for osteosarcoma patients.

## Author Contributions

LY and XH: conceptualization, investigation, methodology, and writing-original draft. ZS: funding acquisition, writing-review and editing. HG, LW, WW, DJ, and WY: software, supervision, and writing-review and editing. All authors have given final approval to this version of the manuscript to be published.

## Conflict of Interest

The authors declare that the research was conducted in the absence of any commercial or financial relationships that could be construed as a potential conflict of interest.

## Publisher’s Note

All claims expressed in this article are solely those of the authors and do not necessarily represent those of their affiliated organizations, or those of the publisher, the editors and the reviewers. Any product that may be evaluated in this article, or claim that may be made by its manufacturer, is not guaranteed or endorsed by the publisher.

## Abbreviations

OS: osteosarcoma
MVBs: multivesicular bodies
ILVs: intraluminal vesicles
ER: endoplasmic reticulum
TGN: trans-Golgi network
BME: bone microenvironment
CAFs: cancer-associated stromal fibroblasts
Tim-3: T cell immunoglobulin and mucin domain 3
EMT: epithelial mesenchymal transition
MSCs: mesenchymal stem/stromal cells
TME: tumor microenvironment
SD-MSCs: stressed mesenchymal stem cells
hBMSCs: human bone marrow mesenchymal stem cells
CDDP: cisplatin
BMSCs: bone mesenchymal stromal cells
ADSCs: adipose tissue-derived mesenchymal stromal cells
BCL6: B cell lymphoma 6
PVT1: plasmacytoma variant translocation 1
CASC15: cancer susceptibility 15
EWSAT1: Ewing sarcoma associated transcript 1
uPA: urokinase plasminogen activator
COLGALT2: collagen beta galactosyl transferase 2
PD-L1: programmed death-ligand 1
COL6A1: Collagen type VI alpha 1
LCP1: lymphocyte cytosolic protein 1
Hic-5: hydrogen peroxide inducible clone 5
TEM: transmission electron microscope
qRT-PCR: quantitative real time polymerase chain reaction
SEM: scanning electron microscope
NTA: nanoparticle tracking analysis
PDAC: pancreatic ductal adenocarcinoma
DC: dendritic cell
CRC: colorectal cancer
Mart1: melanoma-related antigen recognized by T cells
Aex: ascites
GM-CSF: granulocyte-macrophage colony-stimulating factor
PM: plasma membrane
ESCRT: endosomal sorting complex required for transport complex
RAB: Ras-related proteins in brain
ARF6: ADP-ribosylation factor 6
ALIX: ALG2-interacting protein X
TSG101: tumor susceptibility gene101.
